# The complete chloroplast genome of *Eichhornia crassipes* (Pontederiaceae) and phylogeny of commelinids

**DOI:** 10.1080/23802359.2019.1667901

**Published:** 2019-09-23

**Authors:** Qing Ma, Yin Lu

**Affiliations:** College of Biological and Environmental Engineering, Zhejiang Shuren University, Hangzhou, Zhejiang, China

**Keywords:** Commelinids, *Eichhornia crassipes*, chloroplast genome, phylogeny

## Abstract

*Eichhornia crassipes* is a floating aquatic plant native to the American tropics. Here we reported and characterized the first complete chloroplast (cp) genome of *Eichhornia crassipes* and analyzed the phylogenomic relationship of Commelinids based on complete chloroplast sequences. The complete chloroplast genome of *Eichhornia crassipes* has a typical quadripartite structure with a total length of 161,783 bp. A total of 124 genes, including 85 encoding genes, 38 transfer RNA genes, and 8 ribosomal RNA genes were annotated. The phylogenomic study validated the phylogenetic position of *Eichhornia crassipes* and showed that four species from Commelinales form a monophyletic group sister to Zingiberales.

*Eichhornia crassipes* is an invasive aquatic plant native to the American tropics. After introduction into Africa, Asia, Europe, as well as the South Pacific, it has become a naturalized weed beyond control due to its great reproduction capacity (Kriticos and Brunel 2016; Jones et al. 2018). However, recently more and more studies have been focused on *E. crassipes* for its potential in phytoremediation and wastewater restoration, particularly in the rhizofiltration of water containing heavy metals (Yan et al. 2017; De Laet et al. 2019). To further understand its genetic background, here we assembled and characterized the first complete chloroplast genome of *E. crassipes*.

Leaf materials of *E. crassipes* were collected from Hangzhou Botanical Garden, Zhejiang, China. The corresponding specimen was deposited in the herbarium of Zhejiang Shuren University (ZJSRU) (voucher number: Ma20190608). Total DNA was isolated using the DNA Plantzol Reagent (Invitrogen, Carlsbad, CA) and sent to BGI (Shenzhen, Guangdong, China) for next-generation sequencing on the Illumina Hiseq Platform (Illumina, San Diego, CA). The chloroplast genome was assembled using NOVOPlasty (Dierckxsens et al. 2017) combined with GetOrganelle (Jin et al. 2018). Genes and the corresponding coding regions were annotated using Geneious Prime 2019.2 (https://www.geneious.com). The chloroplast genome sequence of *Xiphidium caeruleum* (GenBank accession number: JX08669.1) was used as the reference (Barrett et al. 2013).

The chloroplast genome of *E. crassipes* is 161,783 bp in length with a typical quadripartite structure consisting of a large single copy (LSC) of 89,682 bp, a small single copy (SSC) region of 18,447 bp, and a pair of 26,827 bp inverted repeats (IRs). The GC content of the complete chloroplast genome is 36.5%. The genome contains 124 genes, including 85 encoding genes, 38 transfer RNA genes, and 8 ribosomal RNA genes. The IR regions contain 6 coding sequences (CDSs), 8 tRNA genes, and all the four rRNA genes. The complete chloroplast genome structure, GC content, as well as gene locations of all the 124 genes are almost identical to the previously reported chloroplast genomes of *X. caeruleum* and is largely similar to the chloroplast genomes of species from Commelinales (Barrett et al. 2013; Ma and Liang 2019).

Phylogenomic analysis based on the complete chloroplast genome was performed with the maximum-likelihood (ML) method (Stamatakis 2014) implemented on the CIPRES Science Gateway V.3.3 (Miller et al. 2010). Chloroplast genome sequences of three species from Commelinales, three species from Arecales, 9 species from Poales, and 12 species from Zingiberales were downloaded from the NCBI GenBank. Two species from Asparagales were used as the outgroup. Genome sequences were aligned using MAFFT version 7.0 (Katoh et al. 2017). The chloroplast genome sequences of *E. crassipes* was submitted to GeneBank (accession number: MN480421). The phylogeny revealed that *E. crassipes*, *X. caeruleum*, *Hanguana malayana*, and *Pontederia cordata* from Commelinales form a monophyletic group sister to species from Zingiberales. All the species from Commelinids also form a monophyletic group with high support (Figure 1).

**Figure 1.**
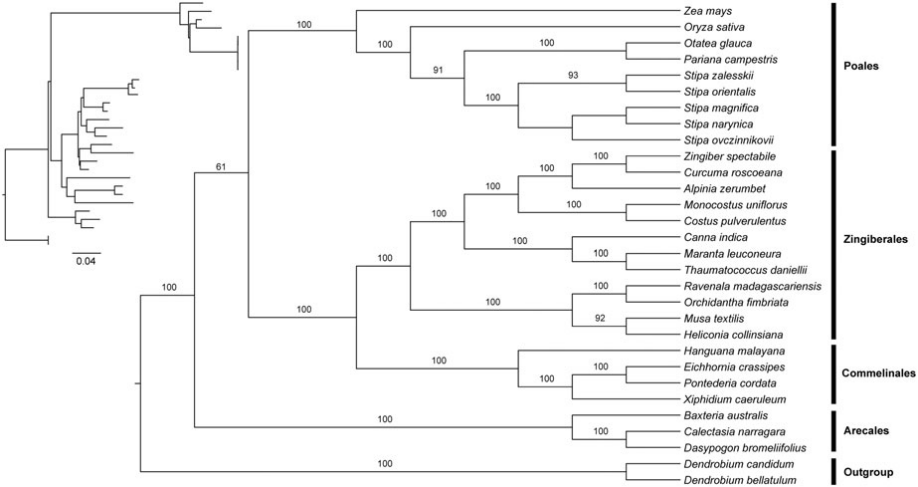
Phylogenetic tree reconstruction of Commelinids using maximum-likelihood (ML) based on whole chloroplast genome sequences. Relative branch lengths are indicated at the top-left corner. Numbers above the lines represent ML bootstrap values. ML bootstrap < 60% was not shown. GenBank accession numbers of all the chloroplast genome used for phylogenomic analysis are shown below: *Hanguana malayana* (NC_029962), *Xiphidium caeruleum* (JX088669), *Pontederia cordata* (MK204378), *Zingiber spectabile* (JX088661), *Curcuma roscoeana* (KF601574), *Alpinia zerumbet* (JX088668), *Monocostus uniflorus* (KF601572), *Costus pulverulentus* (KF601573), *Canna indica* (KF601570), *Maranta leuconeura* (KF601571), *Thaumatococcus daniellii* (KF601575), *Ravenala madagascariensis* (KF601568), *Orchidantha fimbriata* (KF601569), *Musa textilis* (KF601567), *Heliconia collinsiansa* (NC_020362), *Zea mays* (KP966117), *Oryza sativa* (NC_001320), *Otatea glauca* (NC_028631), *Pariana campestris* (NC_027491), *Stipa zalesskii* (NC_037037), *Stipa orientalis* (NC_037033), *Stipa magnifica* (NC_037031), *Stipa narynica* (NC_037032), *Stipa ovczinnikovii* (NC_037034), *Baxteria australis* (NC_029970), *Calectasia narragara* (JX088666), *Dasypogon bromeliifolius* (JX088665), *Dendrobium candidum* (NC_035745), and *Dendrobium bellatulum* (NC_037700).

In conclusion, the complete chloroplast genome of *Eichhornia crassipes* is reported for the first time in this study. It will provide essential resources for future genetic study of this species and also gain insights into evolutionary patterns within Commelinids.
